# MicroSalmon: A Comprehensive, Searchable Resource of Predicted MicroRNA Targets and 3′UTR Cis-Regulatory Elements in the Full-Length Sequenced Atlantic Salmon Transcriptome

**DOI:** 10.3390/ncrna7040061

**Published:** 2021-09-22

**Authors:** Sigmund Ramberg, Rune Andreassen

**Affiliations:** Department of Life Sciences and Health, Faculty of Health Sciences, OsloMet-Oslo Metropolitan University, 0167 Oslo, Norway; sigmundr@oslomet.no

**Keywords:** Atlantic salmon, miRNA, microRNA, cis-regulatory elements, 3′UTR

## Abstract

Complete 3′UTRs unambiguously assigned to specific mRNA isoforms from the Atlantic salmon full-length (FL) transcriptome were collected into a 3′UTRome. miRNA response elements (MREs) and other cis-regulatory motifs were subsequently predicted and assigned to 3′UTRs of all FL-transcripts. The MicroSalmon GitHub repository provides all results. RNAHybrid and sRNAtoolbox tools predicted the MREs. UTRscan and the Teiresias algorithm predicted other 3′UTR cis-acting motifs, both known vertebrate motifs and putative novel motifs. MicroSalmon provides search programs to retrieve all FL-transcripts targeted by a miRNA (median number 1487), all miRNAs targeting an FL-transcript (median number 27), and other cis-acting motifs. As thousands of FL-transcripts may be targets of each miRNA, additional experimental strategies are necessary to reduce the likely true and relevant targets to a number that may be functionally validated. Low-complexity motifs known to affect mRNA decay in vertebrates were over-represented. Many of these were enriched in the terminal end, while purine- or pyrimidine-rich motifs with unknown functions were enriched immediately downstream of the stop codon. Furthermore, several novel complex motifs were over-represented, indicating conservation and putative function. In conclusion, MicroSalmon is an extensive and useful, searchable resource for study of Atlantic salmon transcript regulation by miRNAs and cis-acting 3′UTR motifs.

## 1. Introduction

MicroRNAs (miRNAs) are a group of small non-coding RNAs (sncRNAs) involved in post-transcriptional regulation of almost all cellular processes, ranging from growth, development, and tissue differentiation to maintenance of tissue-specific functions, apoptosis, and immune responses. Because of this, they are of interest to researchers for several reasons, including to gain a better understanding of the molecular mechanisms involved in post-transcriptional gene regulation of various gene networks, as potential biomarkers, and as targets for novel therapeutic approaches [1,2,3].

Mature miRNAs are approximately 20–24 nts in length, and they are processed from miRNA precursors (pre-miRNAs) into pairs of mature miRNAs (a mature miRNA duplex). The two mature miRNAs in the duplex are named 5p and 3p, depending on their relative positions on the pre-miRNA. The duplexed mature miRNAs are loaded into the miRNA-induced silencing complex (miRISC). During this process, one out of the two mature miRNAs (either 5p or 3p) is released from the Argonaute (AGO) proteins and degraded, while the remaining mature miRNA (the guide miRNA) is retained in the miRISC to act as an effector for the RISC to recognize specific target transcripts [3,4,5]. They guide the RISC to their target messenger RNAs (mRNAs) by partial base pairing to target sites usually located in the 3′UTR of the target transcripts. The pairing between the “seed” region, which is the 2–8 nts starting at the 5′ end of a mature miRNA, and the target site in the 3′UTR of the target transcript is particularly important. In this part of the miRNA–target site interaction, there is usually complete complementarity between the miRNA and the target site sequence [4,5,6,7]. The successful recognition of a target transcript by a miRNA results in negative post-transcriptional regulation either by degradation of the target transcript or by translational repression [7]. The 3′UTRs of protein-coding transcripts are the non-coding section of mRNAs that allows regulation of mRNAs by the miRISC pathway. Guided by miRNAs that interact with certain miRNA target sites (miRNA response elements (MREs)) in the 3′UTRs, the miRISC is directed to the target transcripts.

The 3′UTRs also facilitate post-transcriptional regulation through a variety of other cis-regulatory sequences that interact with different trans-acting factors that affect the translation or stability of vertebrate mRNAs [8,9,10]. While some cis-acting motifs in the 3′UTRs have been characterized [11], there has not been any large study aiming to identify such cis-acting elements in Atlantic salmon.

Knowledge of what is important in a successful miRNA–MRE interaction, such as complementarity in the seed sequence and the total free energy of the paired miRNA–MRE sequence, is used in several algorithms that predict whether a certain miRNA is likely to successfully target a given transcript (in silico target predictions) [12]. Some of the most common of such prediction tools, which were all employed in this study, are RNAhybrid [13,14], TargetSpy [15], PITA [16], and miRanda [17]. Such tools are widely used for in silico prediction of target genes. Despite some having been shown to predict a significant number of false positives [18,19], they do provide important information about miRNA–target transcript interactions. By applying these tools, one may identify the subset of a species’ protein-coding genes with 3′UTR sequences that fulfill the MRE criteria for successful interaction with a particular mature miRNA. These prediction tools, thus, identify putative target transcripts of certain miRNAs. The accuracy of these methods depends heavily on access to well-characterized miRNAomes from the species of interest as well as well-characterized 3′UTRs from the complete transcriptome (3′UTRome) [18,19]. 

Recently, mature miRNAs were characterized in Atlantic salmon from 111 samples from different organs and developmental stages (fry, adult) as well as samples from individuals infected with ISAV and IPNV. Individual samples were deep-sequenced, used for miRNA characterization, and finally annotated according to well-described guidelines from miRBase [20,21]. This study complemented the previous characterization study in Atlantic salmon from deep-sequenced samples that were from nine different organs from individuals at the pre-smolt developmental stage, as well as from a 1-day-old individual [22]. All the Atlantic salmon miRNAs in miRBase (http://www.mirbase.org/, accessed on 22 September 2021), the primary online resource for mature miRNAs and their pre-miRNAs, are from these studies [20,22]. Due to these efforts, the miRNAs in Atlantic salmon are among the best characterized among teleosts. The 589 unique mature miRNAs, including mature miRNAs from 17 novel miRNA genes (which so far have only been discovered in Atlantic salmon), are available from Woldemariam et al. [20]. 

Due to a lack of sequence data that spanned the entirety of the transcripts (full-length sequenced mRNAs (FL-mRNAs)), the vast majority of mRNA transcript sequences in Atlantic salmon were until recently predicted using the genome sequence supported by short-read sequencing data [23]. However, retrieving 3′UTR sequences from genome predictions is a dubious approach, and in salmonids, the relatively recent whole genome duplication (SS4R, 80 mya) further complicates the prediction of 3′UTR sequences. Retrieving the correct 3′UTR for a particular paralog would require that all downstream sequences of highly similar paralogs be correctly annotated. This lack of reliable 3′UTR information has likely affected the accuracy of in silico target gene predictions as well as prevented any large-scale investigations of other 3′UTR motifs affecting post-transcriptional regulation in Atlantic salmon. This obstacle was recently removed as the Atlantic salmon transcriptome was characterized applying single-molecule long-read sequencing methods [24]. This work provided functionally annotated high-quality FL-mRNAs for more than 70,000 protein-coding transcripts mapped to more than 23,000 loci. The generation of a dataset containing predictions of all likely mRNA targets for all known Atlantic salmon miRNAs would be of great value to the ongoing research on post-transcriptional regulation of gene expression in Atlantic salmon. A searchable resource with a comprehensive overview of which transcripts contain cis-regulatory motifs known as functionally important elements in vertebrate 3′UTRs would also be a useful resource. The recently available full-length sequenced transcriptome allows for such investigations as the 3′UTRs may be reliably extracted from a given mRNA and, thus, are unambiguously associated with the protein encoded by the CDS [24]. 

While the regulatory motifs associated with transcription of Atlantic salmon genes have been studied [25], studies of motifs associated with the post-transcriptional regulation of transcripts are few [11]. Several studies have carried out target gene predictions using a smaller subset of mature miRNAs and mRNA transcripts as input, e.g., [26,27,28,29] to predict MREs in the input mRNAs. However, due to limited access to high-quality 3′UTR sequences, there have not been any attempts to make a complete prediction analysis of all putative target transcripts in the Atlantic salmon transcriptome by applying the complete Atlantic salmon miRNAome as input. The aim of this study was therefore to extract 3′UTRs from the high-quality, full-length sequenced Atlantic salmon transcriptome and make it publicly available. This 3′UTRome along with a description of the 3′UTR characteristics of Atlantic salmon protein-coding transcripts would itself be a useful resource. The 3′UTRome was then used to identify all putative miRNA target genes in the recently characterized full-length sequenced transcriptome. This resource is made available in a comprehensive and easily searchable dataset of predicted miRNA targets. Finally, an overview of known cis-acting motifs as well as conserved novel and putatively functional motifs discovered in the 3′UTRs of the recently full-length sequenced Atlantic salmon transcriptome was included in the MicroSalmon resource.

## 2. Results

### 2.1. A Searchable 3′UTR Resource with miRNA Targets and 3′UTR Regulatory Motifs 

#### 2.1.1. A Comprehensive 3′UTR Resource Extracted from FL-mRNAs

The 3′UTR was retrieved from each of the transcripts in the TSA submission GIYK01000000 [24], as described in the Materials and Methods section. The complete set of these extracted 3′UTR sequences (the 3′UTRome) is included in the MicroSalmon GitHub repository at http://github.com/AndreassenLab/MicroSalmon/ (Uploaded 13 September 2021). The file containing the Atlantic salmon 3′UTRome resource, named mRNA_3UTR.fasta, is given in the DATA folder. The Atlantic salmon 3′UTRome includes Genbank accession numbers for each of the 3′UTRs so that they are easily associated with their complete FL-mRNA in the full-length sequenced transcriptome [24]. The SeqID is also given for each of the 3′UTRs. The SeqID annotation groups together putative splice variants, and it also indicates the species genome having the best match to the transcript in the Cupcake clustering and SQANTI analysis by Ramberg et al. [24].

**Figure 1:**
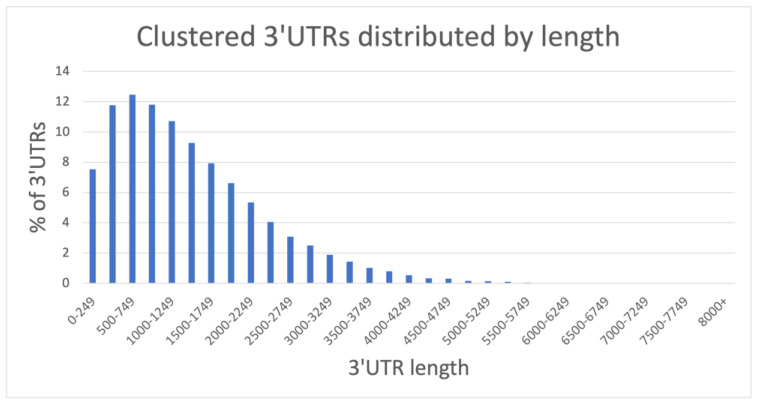
Distribution of 3′UTR lengths in the non-redundant 3′UTRome.

A large number of transcripts are splice variants, and consequently, such splice variants often share the same 3′UTR sequence. The identical 3′UTR sequences from such splice variants were clustered using CD-HIT (see the Materials and Methods section) to avoid including several thousands of replicates of the same 3′UTRs in our analysis. This clustering process reduced the number of input UTR sequences from 71,461 to a non-redundant set of 43,305 3′UTRs. The non-redundant set was, thus, from different loci or from same loci but with differently spliced 3′UTRs. The size distribution of the 3′UTRs in the non-redundant set is shown in Figure 1. The sequences in the reduced 3′UTR set ranged from 11 to 8164 nts, with a median length of 1146 nts.

Following clustering of redundant 3′UTR sequences, the nucleotide distribution of the 3′UTRs was investigated. There was an overabundance of thymine and adenine, making up 30.7% and 28.1% of the sequences, respectively, while guanine made up 20.7% and cytosine made up 20.5%. This is similar to the findings of Andreassen et al. [11]. The observed proportion of bases was used in the estimates of the expected random occurrence of different sequence motifs in the 3′UTRome (See Section 2.4 and the Materials and Methods section). 

#### 2.1.2. MicroSalmon: A Searchable Resource with In Silico Predicted miRNA Targets

**Figure 2:**
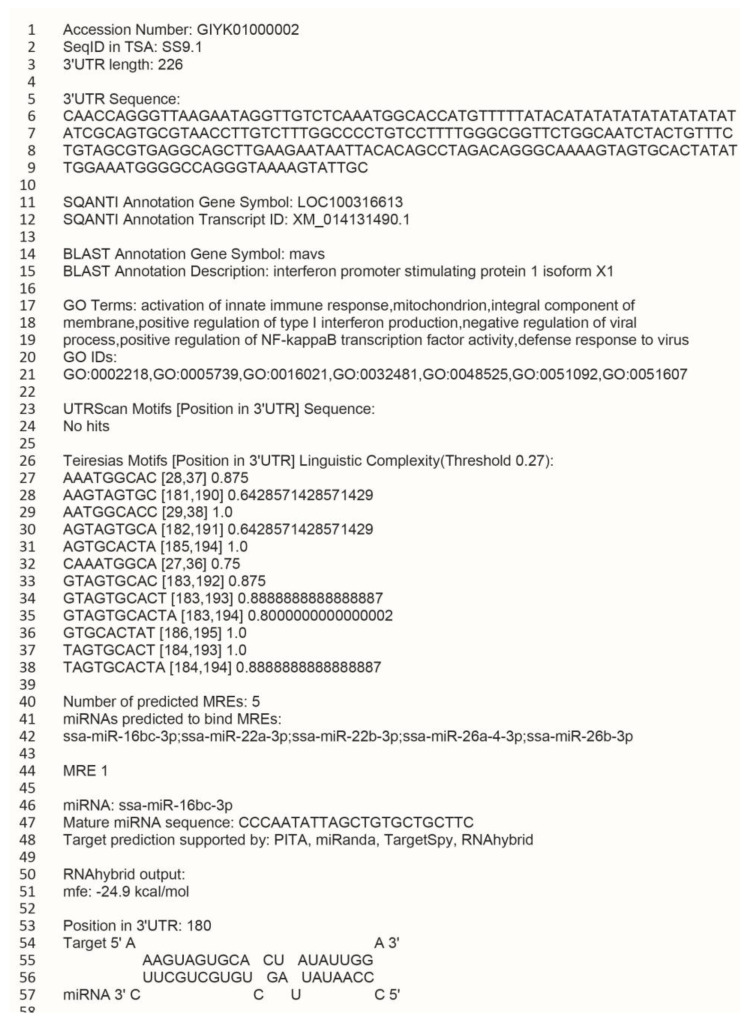
The simple text file output from a search using the transcript accession number as input.

The list of miRNA targets supported by RNAhybrid and at least two of the miRNAconTarget tools (PITA, miRanda, TargetSpy) is included in the MicroSalmon GitHub DATA folder, in the file named RNAhybrid_plus_2 (http://github.com/AndreassenLab/MicroSalmon/, uploaded 13 September 2021). The two input files for the in silico analysis, all Atlantic salmon miRNAs from Woldemariam et al. [20] and the 3′UTRome (Section 2.1.1), are also given in the data folder and named miRNAome and mRNA_3UTR, respectively. Additionally, Python3 scripts are included that allow users to search for a specific miRNA or a list of miRNAs to reveal their predicted target transcripts. Likewise, by using transcript annotation (Genbank accession number or seqid), their gene symbols, or Gene Ontology (GO) terms (from the mRNA_3UTR or SQANTI_OmicsBox_Annotation files in the DATA folder) as input, searches may identify miRNAs predicted to target the input. Detailed instructions on how to use the scripts are given in Section 4.2.3 and in the readme file in the GitHub repository. An example of the search outputs that are uploaded in the OUTPUT folder in simple text format is shown in Figure 2. The example in Figure 2 is from a search where the transcription accession number was used as input (GIYK01000002). Any such search will give input information in the header section (lines 1–3 in the figure), in this case the transcript Genbank accession number, SeqID in the TSA database, and length of the 3′UTR. Lines 5–38 provide additional information about the transcript, including the complete 3′UTR sequence (lines 5–9), additional annotation on the transcript retrieved from the full-length transcriptome [24] (lines 11–21), and results from searches of other known or predicted cis-regulatory motifs in the 3′UTR in lines 23–38 (See also Section 2.1.3). The results from the miRNA target prediction follows this summary information. Line 40 lists the number of MREs predicted in this particular 3′UTR, while line 42 lists which mature miRNAs that were predicted to target the transcript. In Figure 2, there are five mature miRNAs predicted to bind an MRE in this transcript. The remaining output gives detailed information about each of the mature miRNAs and the MRE(s) that were targeted (only the first is shown in Figure 2). This includes a graphic illustration of the miRNA–MRE interaction, the minimum free energy, and the target prediction tools that supported this miRNA–MRE interaction. 

If using one of the other search scripts that take a miRNA, gene symbol, or GO ID as the search query, the output will additionally contain a summary data section below the header, showing an aggregate of the search output for all mRNAs associated with the query, followed by a separate section, as described above, for each mRNA. Supplementary Files S1 and S2 shows complete simple text file outputs when applying an mRNA accession number (GIYK01000002) or a mature miRNA name (ssa-miR-10d-3p) as input. The output file may be simplified by modifying the default search to remove some of the sections. All such modifications of the default search scripts is described in detail in the Materials and Methods section and in the help file for each search script given in the GitHub repository. 

This resource and the additional scripts, thus, provide information about any miRNAs predicted to target an FL-transcript along with detailed information about the miRNA–target interaction (MRE). The reverse is also possible—to search for all transcripts targeted by a particular miRNA or a set of miRNAs.

**Figure 3:**
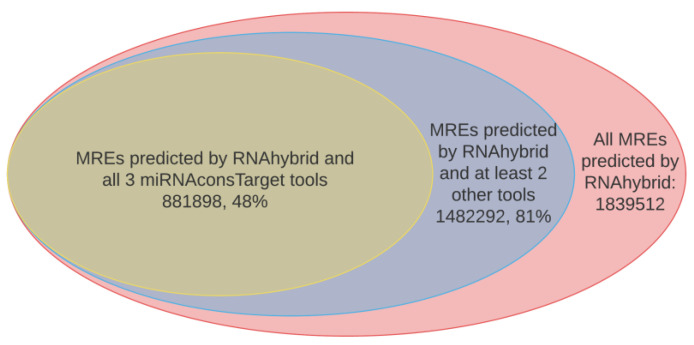
Number of MREs predicted by RNAhybrid (red), RNAhybrid and at least two other of the miRNAconsTarget tools (blue), or RNAhybrid and all three miRNAconsTarget tools (yellow).

A total of 1,839,512 MREs were predicted by RNAhybrid, which was used as our primary target prediction tool (see the Materials and Methods section; Figure 3). Aiming to reduce the number of false-positive targets, three more target prediction tools (PITA, miRanda, and TargetSpy) were used to increase the likelihood that a particular MRE was not a false-positive result (see the Materials and Methods section). Applying a lower “match threshold” of at least two additional tools predicting the same target transcript for a particular miRNA, the number of MREs was reduced to 1,482,292. This showed that introducing a more stringent match criterion removed about 19% of the MREs predicted by RNAhybrid alone. Applying a still more conservative match criterion for MREs by demanding that they should be predicted by both RNAhybrid and all of the three other tools would have reduced the number of predicted MREs to 881,898. This is approximately 48% of those MREs predicted by RNAhybrid alone, revealing that only about half of the MREs predicted by RNAhybrid were supported by all four tools. Although applying such stringent match criteria could further remove false positives, it would also increase the likelihood that many true targets would not be reported. Thus, all the targets predicted by RNAhybrid and at least two more of the prediction tools were included in the MicroSalmon resource. The search output does, however, show tools that supported each of the MRE predictions, and this allows further manual filtering of which targets to include, if desired.

#### 2.1.3. The MicroSalmon Resource Also Includes Other Predicted Cis-Regulatory Motifs

The MicroSalmon GitHub repository also contains restructured output from cis-regulatory element prediction using UTRscan [8]. All identified 3′UTR motifs can be listed for each FL-mRNA in the search outputs, and the complete file providing all 3′UTRs with a UTRscan motif is given in the DATA folder of the MicroSalmon repository (uscan_output). Finally, putative cis-regulatory motifs were identified by their over-representation in the 3′UTRs (Teiresias algorithm, see the Materials and Methods section). Results from this analysis were also included in the repository. These motifs, annotated as Teiresias Motifs (Figure 2), are also given as additional information in all search outputs for each of the transcripts. By default, the search outputs only include high-complexity motifs but have the option to include low-complexity motifs by setting a lower minimum complexity threshold.

### 2.2. Results from In Silico miRNA Target Prediction Analysis

Each of the 589 mature miRNAs in the current Atlantic salmon miRNAome [20] had at least one hit within the 3′UTRome. Ssa-miR-181e-5p was the mature miRNA having the largest number of predicted target mRNAs, putatively targeting 12,354 FL-mRNAs, while ssa-miR-10d-3p had the fewest, with 196 predicted targets. Most mature miRNAs had several thousand hits in the FL-transcriptome, and the median number of FL-mRNA target transcripts was 3778. However, a large number of the 3′UTRs are from splice variants (estimated to be an average of three per locus [24]), and 3′UTRs from splice variants originating from the same locus were usually targeted by the same miRNA. In addition, a given 3′UTR may have several MREs for the same or different miRNAs. For these reasons, the number of different unique genes targeted by a given miRNA was much lower than the number of FL-mRNA targeted transcripts. For example, ssa-miR-181e-5p, the miRNA with the highest number of FL-mRNA targets, had only 4516 unique target genes, about a 2.5-fold reduction from the number of target transcripts. The difference in the number of FL-mRNAs compared to different (unique) target genes was also reflected in the median number of unique genes targeted by a miRNA, with a median of 1487 different genes, while the median of FL-mRNAs was 3778 (which included splice variants).

The number of predicted miRNA–MRE interactions was far in excess (>20×) of the number of mRNAs in the 3′UTRome. The fact that many transcripts were targeted by multiple, often more than 100 miRNAs was the reason for this large difference between the number of transcripts and MREs. The transcript GIYK01025461, annotated as a novel isoform of the gene tmp1, had the highest number of targeting miRNAs, with 194 mature miRNAs matching an MRE in the 3′UTR of this transcript. However, a total of 5786 transcripts (from 1637 different genes) had no predicted MREs at all. The median number of targeting miRNAs for a transcript was 27. The results agree with findings in other species that one miRNA may regulate many transcripts, while one transcript may be regulated by many miRNAs.

### 2.3. Identification of Known Cis-Regulatory Elements

A total of 32,333 transcripts, representing 10,939 unique genes, had at least one UTRscan predicted motif in their 3′UTR. Nineteen of the 3′UTR cis-regulatory motifs in UTRdb were detected. The distribution, description, and references for these motifs are shown in Table 1. The most common remaining motifs were K-Box, identified in 14,341 different transcripts, followed by BRD-Box, identified in 6635 different transcripts. The proneural box feature was only identified in one transcript, SS3916.1, which was annotated as originating from a novel gene with unknown functions (SQANTI and OmicsBox protein BLAST analysis [24]).

To further explore whether the predicted UTRscan motifs were likely to be true cis-regulatory motifs in Atlantic salmon, we carried out manual annotation, Gene Ontology (GO) analysis, and gene pathway enrichment analysis. The manual annotation of the 138 transcripts revealed that they were from 43 different genes, not 49 as anticipated from the loci count from Ramberg et al. [24] or 54 as anticipated from the GenBank annotation. The expectation was that if IRE was a true functional cis-acting motif in Atlantic salmon, it would be present in transcripts associated with Gene Ontology (GO) terms or gene pathways associated with iron transport or metabolism [32,33,34]. The complete set of GO annotations from Ramberg et al. [24] was retrieved for all these transcripts, and gene symbols were added by manual annotation (See the Materials and Methods section and Supplementary SFile 3). After trimming away redundant gene symbols, the final set (Supplementary File S3) was used as input in the enrichment analysis using Enrichr [83,84,85]. 

The results from exploring the function of the transcripts with the iron-responsive element revealed that 9 of the 43 genes had Gene Ontology (GO) annotations or were part of gene pathways involved in iron binding, transport, or metabolism. One gene with a generic gene ID (LOC106613912) was manually annotated as QSOX1, a gene previously described as an iron-responsive tissue-remodeling factor regulated by the IRE motif [86,87]. Another of the genes with a generic ID (LOC106574427) was manually annotated as steap3, which was involved in pathways relating to iron metabolism (Enrichr analysis). Furthermore, the manual annotation revealed that two of the genes were paralogs of the transferrin receptor protein 1 (trfc). The nine genes are shown in Table 2. Together, the predicted functions of these genes all agreed with the IRE motif being a true cis-acting regulatory motif in these transcripts.

### 2.4. Discovery of Novel Putative Functional Cis-Motifs by Their Over-Representation in the 3′UTRome

Using the Teiresias algorithm, a total of 604 motifs were identified that appeared in more than five times as many transcripts as would be expected if they were randomly distributed (see the Materials and Methods section). The distribution of the number of over-represented motifs ranging in size from 7 to 16 nts is shown in Table 3. Eighty-two of these motifs contained heptamers complementary to the seed of 13 Atlantic salmon miRNAs (shown in Supplementary File S4). This opens the possibility that their over-representation was the result of being part of MREs that have been selectively retained in the 3′UTRs. However, the motifs with target sequences matching the ssa-miR-737–5p seed, which consists of a heptamer of thymine residues, were A-rich homopolymers. It is likely that these motifs are over-represented due to matching other functional motifs skewed toward the end of the 3′UTR (see below).

A complete overview of all 604 over-represented motifs is given in Supplementary File S5. Although some low-complexity motifs may be functional cis-regulatory motifs, the over-representation of low-complexity motifs containing, e.g., homopolymers or short tandem repeats (STRs), does itself not indicate that they are conserved functional motifs. However, several of the over-represented low-complexity motifs detected in the Atlantic salmon 3′UTRome have indeed been shown to be cis-acting 3′UTR motifs in vertebrates. There were, e.g., 151 over-represented motifs that had either the ARE-motif (ATTTA) [88], the CPE-consensus motif (TTTTATT) [89], U-rich motifs (TTTNTTT), or the destabilizing motif CCTCCNC [90]. These cis-acting motifs all have in common with the MREs interacting with miRISC that they affect the stability of mRNAs in certain tissues and/or developmental stages [88,89,90]. There were also 18 over-represented motifs that had the PAS-motif (AATAAA) [11,91]. The PAS-motif function is to initiate poly-adenylation from a certain position in the 3′UTR of the precursor mRNA. This motif is also supported by upstream or downstream T (U)-rich auxiliary sequences [92]. This is likely the reason why many U-rich motifs were over-represented (see below). 

The location of the over-represented sequences within the 3′UTR was mapped for all motifs (Supplementary File S5). This revealed that 124 of the motifs were significantly enriched within the 10% nts after the stop codon, while 107 motifs were significantly enriched in the 10% nts at the 3′ end of the 3′UTRs. Moreover, those located immediately downstream of the stop codon were either pyrimidine-rich (C/T) or purine-rich (A/G) motifs. The 107 located close to the 3′ end were mostly T (U)-rich motifs. Many of these were matches with the U-rich motifs reported in zebrafish [90], or they could be the auxiliary T (U)-rich sequences close to the terminal end that support PAS function [92,93]. The A-rich motifs matching the ssa-miR-737-5p were also skewed toward the 3′ end (Supplementary File S5). However, the over-representation of such motifs in this particular location was likely due to their match with the PAS rather than being ssa-miR-737-5p targets.

To further explore the complex motifs, additional filtering was applied on the motifs identified by the Teiresias algorithm (see the Materials and Methods section) to remove those consisting of low-complexity sequences. An over-representation of such complex motifs could be a consequence of conservation by purifying selection, as expected if they were functional cis-acting motifs. This additional filtering showed there were 82 complex motifs among those initially identified by the Teiresias algorithm (Supplementary File S5). Still, 14 of these had known seed targets as part of their motif (Table 3). In contrast to the low-complexity motifs, the complex motifs were, in general, distributed equally over the 3′UTRs.

## 3. Discussion

### 3.1. The Accuracy and Limitations of In Silico miRNA Target Prediction

In silico miRNA target prediction is still an imperfect analysis method. There are a variety of different algorithms used by different in silico prediction tools [12,18,19,94,95], all being prone to produce both false positives and false negatives [19]. RNAhybrid was applied as our primary algorithm. The program identifies the potential target sites in the 3′UTRs based on the absolute seed match and the most favorable free energy between the miRNA–mRNA hybrids. The advantage of RNAhybrid is that it allows the user to define input miRNA and target regions, as well as manipulate several additional settings important for the target predictions (e.g., G:U in the seed match, free-energy cutoff, helix constraint, and maximal loop size). Applying this as the primary in silico prediction tool, the filter criteria for identifying MREs could be specified in an unambiguous manner, and the outputs could include a clear visualization of the binding interaction in plaintext format. In addition, the final MREs predicted in our in silico analysis were based on matches in at least two more prediction tools from the miRNAconsTargets package (TargetSpy [15], PITA [16], and miRanda [17]) that used additional criteria such as the conservation level and accessibility to the binding site in the 3′UTR to identify MREs [95]. Applying several slightly different prediction tools that complement each other might be a way to reduce the number of false positives in the resulting final set [95]. Support by RNAhybrid and at least two other tools was therefore chosen as our criterion for inclusion in the final dataset. Reporting only MREs identified by all tools could, in our view, be a too restrictive approach and lead to loss of true MREs (false negatives). However, all outputs list the prediction tools supporting any MRE, and if the user prefers a more conservative approach, they may choose to include only the targets predicted by all four tools. 

Despite the inclusion of both false positives and false negatives in their results, the in silico prediction tools applied in this study have been shown to predict a number of target mRNAs that later have been validated by experimental methods in many species (https://mirtarbase.cuhk.edu.cn/~miRTarBase/miRTarBase_2019/php/index.php, accessed on 22 September 2021). Among the validated conserved targets in other vertebrates are also target mRNAs that were predicted in our Atlantic salmon resource. Some examples are miR-221 targeting cyclin dependent kinase inhibitor 1B (mirtid = MIRT000137), miR-301a targeting BTG1 (mirtid = MIRT734312), and miR-101a targeting SOX9 (mirtid = MIRT053036).

The distribution of predicted MREs for each miRNA in the final dataset, with a median of 3778 transcripts targeted by a miRNA, clearly illustrates that relying on in silico target prediction alone will produce a high number of putative target transcripts for each miRNA. When studying miRNAs affecting certain conditions (i.e., immune response or tissue functions), such in silico predictions would not be useful if being the only approach to pointing out the target transcripts of interest, as they will result in a large number of predicted targets that are not relevant to the study. A common approach is, however, to identify both differentially expressed miRNAs (DE miRNAs) and differentially expressed genes (DEGs) in the same materials (or better yet, perform protein expression analysis). Then the candidate targets to include from the in silico predictions could be limited to those changing their expression when analyzed in the same conditions. Alternatively, the candidate targets may be filtered by their functional annotation to retain those known as important to the condition studied. Applying such experimental strategies together with the predicted targets in the MicroSalmon resource, a smaller but complete set of relevant target transcripts may be identified. Ultimately, such miRNA–mRNA interactions must be verified by experimental validation approaches [96,97,98]. We are presently developing functional assays for further functional validation studies in salmon cell lines. Information about such validated interactions will be included in future updates of our MicroSalmon repository. 

### 3.2. Identification of Known and Novel Cis-Regulatory Elements Greatly Expands the Knowledge of Transcript Regulation in Atlantic Salmon

The existence of a functional annotation for the transcriptome explored in this study (Ramberg et al. [24]) provided a means to explore whether the predicted cis-regulatory motifs are true functional elements. Based on the hypothesis that transcripts under the control of the same cis-acting factors likely have related functions, we identified functions associated with the individual transcripts that had the iron-responsive element in their 3′UTRs. The Gene Ontology and gene symbol annotation for each was retrieved using the search scripts included in the MicroSalmon GitHub repository. Since all the transcripts were already functionally annotated [24], we could identify 9 of the 43 genes with IRE motifs as associated with iron transport or metabolism. This indicates that IRE elements are indeed cis-acting motifs in Atlantic salmon and open for the possibility that the transcripts associated with the other 34 genes are similarly regulated by trans-acting factors binding this motif.

Many of the transcripts are simply assigned generic gene IDs (such as LOC or GSONMT IDs) in GenBank in lieu of descriptive gene symbols). These generic IDs cannot be used in pathway enrichment analysis such as Enrichr. However, through manual annotation, we assigned gene symbols to all the generic IDs. This allowed additional pathway analysis that confirmed that several of the transcripts are involved in iron transport or metabolism. Carrying out pathway analysis, thus, highlighted the importance of the GO annotation of the CDS in the FL-transcriptome [24]. We believe that the presently added annotation of cis-acting motifs in the 3′UTRome will be similarly useful when elucidating the function and regulation of these transcripts. This also demonstrated that the current gene annotation of the Atlantic salmon genome is limited and emphasizes the importance of continued focus on improving the functional annotation of animal genomes [99].

Since the cis-regulatory motifs in the UTRdb are unlikely to be comprehensive, especially for species whose genetics are still being explored, we decided that annotation of over-represented motifs in the 3′UTRs would be of interest. Similar studies have been carried out in humans, identifying known functional cis-acting motifs among the over-represented sequences [100,101]. 

**Figure 4:**
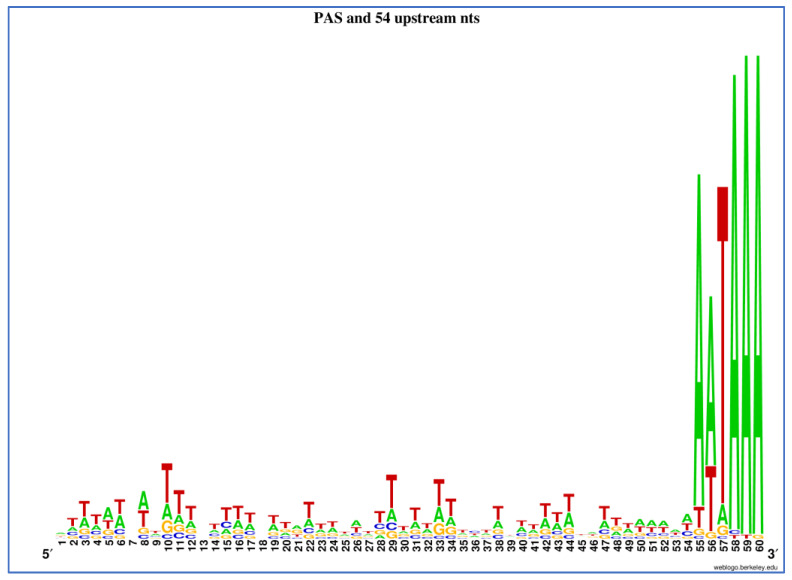
The PAS motif and the immediate nucleotides upstream of PAS in 100 transcripts.

Examination of the over-represented predicted motifs that were 7 nts or longer revealed that many had low complexity. Low-complexity regions, such as tandem repeats or homopolymers, are known to frequently change in length and expand to motifs of 7 nts or more due to the mutation mechanisms acting on such low-complexity regions [102,103]. A high abundance of such motifs does therefore not per se support that they are functional motifs. Despite this, some of the over-represented low-complexity motifs were indeed known to have regulatory functions in vertebrates by affecting mRNA stability in certain tissues or developmental stages [88,89,90]. The skewed distribution of their locations in the 3′UTR sequences also supported the predicted function, e.g., as auxiliary motifs that support PAS function (Supplementary File S5). Figure 4 illustrates the PAS motif and the 54 nts immediately upstream of the PAS motif in 100 randomly chosen transcripts (Supplementary File S6). About one-third of these were 3′UTRs from the same locus but with an alternative use of PAS. This seems to be a quite common mechanism in Atlantic salmon, which may lead to alternative regulation of transcripts if cis-acting motifs are left out in the shorter isoforms. The nucleotide distribution in Figure 4 clearly supports that there were U-rich sequences upstream of the PAS motif but not enriched at a particular location within the region immediate upstream of the PAS motif.

Some over-represented motifs also contained heptamers that were complementary to miRNA seed sequences. This has also been observed previously in similar studies [11,100,101]. These motifs being part of MREs could be the reason why these motifs were over-represented. 

In light of low-complexity motifs being prone to be over-represented purely by high mutation rates acting on such motifs, we decided to filter the over-represented motifs by their linguistic complexity, while still retaining the option to retrieve low-complexity motifs from the dataset. The resulting 82 over-represented and high-complexity motifs could not have their relatively high abundance in the 3′UTRome explained by mutation mechanisms that act on low-complexity regions, such as tandem repeats or homopolymers. Rather they could be the result of conservation through purifying selection. The fact that many of the over-represented low-complexity motifs have been shown to be cis-acting regulatory motifs lends credence to the same being true for these over-represented novel complex motifs However, whether they represent true cis-acting regulatory motifs will ultimately have to be verified by further experimental studies.

### 3.3. The MicroSalmon Repistory

Large-scale target prediction for the miRNAs of a species is not a novel concept. miRDB is a target prediction resource spanning five model species, featuring an interactive search interface, and has been cited over 700 times in the 6 years since its publication [104,105]. This demonstrates that these are important resources for the research community. MicroTrout [106] is a similar prediction framework in a closely related species, *Onchorhynchus mykiss*. It enables search and filtering by use of Excel entries, where each column can be filtered by content separately, rather than plaintext input and output, as is used in MicroSalmon. While miRDB and MicroTrout also allow for identification of associated gene and GO annotation for miRNAs, MicroSalmon also includes information about other cis-regulatory elements in the 3′UTRs.

The inclusion of both MREs and predicted cis-regulatory motifs forms an important contribution to the functional annotation of Atlantic salmon genes. This is the first resource of this kind, as the complete 3′UTRs are unambiguously identified by using error-corrected long-read transcript sequencing. This allows for detection of 3′UTR splice variants that are potentially differently regulated by miRNAs. MicroSalmon may be used as a starting point to retrieve cis-acting motifs (both MREs and others) in FL-transcripts of interest, which can be validated by experimental methods. Furthermore, it may serve as a reference for targeted HTS studies to identify genetic variation that affect the function of such elements [91,101,107,108]. MicroSalmon also represents a resource for the discovery of transcript variants that are differently regulated due to alternative polyadenylation of the 3′UTRs. Additionally, as these types of comprehensive prediction analyses are made available from full-length sequenced transcriptomes in more teleosts [109], identification of true MREs can be further improved by comparative studies that identify conserved gene–miRNA interactions across species.

The increase in reliable, full-length sequenced 3′UTRs from the FL-transcriptome has increased the number of Atlantic salmon 3′UTRs by about 100× compared to the ones available until now in GenBank (3474) or salmonid 3′UTRs in UTRdb (4459) [8]. Consequently, the number of targets that were predicted in MicroSalmon has also increased. A comparison with our study of miRNAs associated with SAV infection [27] is given in Supplementary File S7. This file shows the genes targeted by the differentially expressed miRNAs associated with SAV when using the GenBank-derived 3′UTRs versus the target genes predicted if applying the 3′UTRs from the FL-transcriptome. The comparison shows a greater than 100× fold increase in putative targets identified. Furthermore, about 15% of the NM sequences do not match any of the FL-transcripts. While it is possible that some of these genes were not expressed and thus not identified in the FL-transcriptome study, it is also likely that some of the NM sequences, mostly made from alignments of EST sequences, are erroneous. Likewise, some of the predicted target genes in the SAV study that are also present in the FL-transcriptome (BLAST local sequence homology >90%) were not predicted as targets in the MicroSalmon repository. Again, this is likely due to small sequence differences between the NM entries and the full-length sequenced 3′UTRs. In conclusion, the MicroSalmon repository leads to a huge increase in putative target genes based on the analysis of high-quality full-length sequenced 3′UTRs.

All files in MicroSalmon are made publicly available as a GitHub repository, as this allows for the simple inclusion of scripts for searching through the results. Making MicroSalmon available as a GitHub repository also allows for easily updating any part of the dataset when there are future improvements of the Atlantic salmon genome annotation and assembly, or the inclusion of results from analysis of the 3′UTRs of additional FL-transcripts. The prediction results, annotation, and sequence information are all in transparent machine-readable plaintext format and are published under a license that allows for their use in further development of supplementary scripts or integration of other data, as desired. Databases only available through a browser-based search interface often become permanently inaccessible when a domain at an institution is changed or the project runs out of funding. In contrast, the GitHub repository represents a persistent resource for these data and future iterations, not affected by such practical matters.

In conclusion, we believe that MicroSalmon in its current form will be a useful resource for researchers studying Atlantic salmon miRNAs and transcript regulation by cis-acting 3′UTR motifs. 

## 4. Materials and Methods

### 4.1. Materials

The 3′UTR sequences used for target prediction analysis were retrieved from 71,461 FL-transcripts generated by single-molecule long-read sequenced mRNAs that were error-corrected with Illumina reads [24]. The FL-transcriptome is available from the NCBI under TSA accession number GIYK01000000. The functional annotation of transcripts in this FL-transcriptome is given in Supplementary File S1 in Ramberg et al. [24]. Information about the sample materials used to generate the FL-transcriptome is given in Ramberg et al. and Shwe et al. [24,28].

The set of mature miRNA sequences used for target prediction analysis was the 589 mature miRNAs characterized in Woldemariam et al. and Andreassen et al. [20,22]. These are also given in the file miRNAome.fa in the DATA folder of the MicroSalmon GitHub repository at http://github.com/AndreassenLab/MicroSalmon/ (Uploaded 13 September 2021).

### 4.2. Methods

#### 4.2.1. In Silico Prediction of miRNA Targets

The CDS for each transcript was predicted using TransDecoder, as described by Ramberg et al. [24]. The complete set of Atlantic salmon 3′UTR sequences was extracted from the FL-transcriptome using a Python script, which retrieved the sequence downstream of the predicted stop codon in each of the FL-transcripts (3′UTRome). Forty-four of the transcripts had 3′UTRs shorter than 11 bp, and these were the only FL-transcripts whose 3′UTRs were not included in the 3′UTRome.

To avoid repeating analysis of identical sequences (those stemming from isoforms of the same 3′UTR sequence) and to minimize the computational load of target prediction, the 3′UTR sequences were clustered using CD-HIT 4.8.1 [110,111] into a non-redundant 3′UTRome. The following parameters were applied: global sequence identity, identity cutoff 97%, length difference cutoff 95%, and hard length difference cutoff 30 bp. Sequences that clustered together using these parameters were considered identical for the purposes of target prediction. A single representative sequence from each cluster, as well as all singleton 3′UTRs, was used in the target prediction analysis. 

Four different target gene prediction tools were used. RNAhybrid [13,14] version 2.1.2 was used with the following parameters: helix constraint 2–8, no limitation on the lengths of UTRs, max. internal loop size 9, max. bulge loop size 8, energy cutoff -18 kcal/mol, and no G:U pairings allowed in the RNAhybrid sections that included the seed. A custom-made Python script was used to retrieve only hits that contained no G:U in the seed region, since this functionality was not included in the stand-alone version of the software. The other three programs were TargetSpy [15], PITA [16], and miRanda [17]. They were all run as part of the software miRNAconsTargets, which is included in the version of the sRNAtoolbox VM from 11/05/19 [112].

Following the analysis with these four tools, an in-house Python script was used to identify target mRNAs that had been predicted by both RNAhybrid and at least 2 of the 3 miRNAconTargets tools.

#### 4.2.2. Identification of Putative 3′UTR Cis-Regulatory Elements

Known 3′UTR regulatory motifs were identified in the 3′UTRome using the web-based UTRScan tool [8]. Due to the file size restrictions inherent in this application, the input 3′UTRome fasta file was split into 9 smaller files with the UNIX split program with the parameter −l 10000. Following analysis, the results were concatenated after trimming leading and tailing text to facilitate further analysis and structuring of results.

The gene symbols and Gene Ontology annotations for transcripts annotated as containing the motif IRE were retrieved from the annotation results of the FL-transcriptome in Ramberg et al. [24]. Since many of the gene symbols from GenBank were generic IDs such as LOC and GSONMT IDs, the genes were also manually annotated with gene symbols to allow for enrichment analysis. The manual annotation was carried out by searching each gene identifier in the NCBI Gene database and UniProt, to replace the generic IDs with gene symbols. In cases where this was not successful, the coding sequence (as annotated in Ramberg et al. [24]) was used as input for BLAST searches against RefSeq, and the gene symbol for the top scoring transcript was used. After removing duplicate gene symbols, the final set (Supplementary File S3) was used as input for pathway enrichment analysis using Enrichr [83,84,85].

Teiresias v0.9.1 [113] with the parameters -w7 -l7 -k1000 -p -v was used to identify over-represented motifs that were 7 nts long. Convolution of these short over-represented motifs combined them when they appeared sequentially, if this still resulted in sufficient support for the longer motifs. This ensured that the output motifs were maximized, meaning they were only retained if they were still over-represented when not part of a longer over-represented motif. An in-house Python script was used to filter the results further, keeping only the motifs appearing in at least 5 times as many UTR sequences as would be expected by chance. The expected number of sequences was calculated using the following Equation (1):

*E = N*(1 − (1 − *P*)*^L−K^*^+1^), (1)

where *E* is the expected number of sequences, *N* is the number of sequences in the reduced UTRome (43305), and *P* is the probability of a motif appearing by chance in a sequence of its length, calculated by multiplying the frequencies of each of the nucleotides. The frequencies of each of the nucleotides were calculated from the reduced 3′UTRome. *L* is the average length of the sequences in the reduced 3′UTRome (1335). *K* is the length of the motif. 

To remove low-complexity sequences deemed unlikely to be functional, the over-represented motifs were additionally filtered by their Trifonov linguistic complexity *CT* [114,115], which is calculated as

(2) $$CT=\overset{N}{\underset{i=1}{\prod }}\left(\frac{V_{i}}{V_{\max i}}\right),$$

where *N* is the length of the motif, *V_i_* is the number of unique substrings of length *i*, and *V*_max*i*_ is calculated as

(3) $$V_{\max i}=\min\left(K^{i},N-i+1\right),$$

where *K* is the alphabet size (4 in the case of DNA), and the other parameters are as in Equation (2). A *CT* value of 0.27 was applied as a threshold, and motifs with *CT* > 0.27 were included in our default search.

The PAS motif and the 54 nts upstream of the PAS motif were retrieved from 100 random transcripts (Supplementary File S6) and were used as input in Weblogo (https://weblogo.berkeley.edu/logo.cgi, accessed on 21 September 2021) to illustrate the occurrence of the different nucleotides in the sequence immediately upstream of the PAS motif. 

#### 4.2.3. The MicroSalmon GitHub Repository

Following reformatting of the output from all analyses (Section 4.2.1 and Section 4.2.2), the results were published in the MicroSalmon GitHub repository in the DATA folder, along with a set of four Python3 search scripts, designed to help finding connected results from the separate analyses. The scripts, miRNA_Search.py, mRNA_Search.py, GO_ID_Search.py, and Gene_Symbol_Search.py, have no external dependencies beyond Python3 and can be used from a command-line interface by calling the scripts using Python and providing the arguments on the command line, as described in the readme file. It is also possible to run all the scripts without the command line, e.g., by running the scripts through the Python IDLE interface. If run in this manner, or without otherwise providing command-line arguments, the scripts will give users the option to add arguments by typing them in the provided field as one would on the command line. For each script, the search terms (one or more miRNAs, mRNAs, GO IDs, or gene symbols) can be provided either directly as an argument using the flag -q, as a list of space-separated values starting and ending with quotation marks, or as a file provided with the flag -I, containing one search term per line. All search results are placed in the folder OUTPUT. The script mRNA_Search.py also has the possible argument -s, allowing users to search using a SeqID as the input rather than an accession number. The SeqID provides information about which transcripts are likely isoforms of each other and also indicates the source species for any SQANTI-based annotation. The SeqIDs were defined in our previous paper based on the Cupcake classification scheme. It begins with a two-letter prefix indicating which genome (or lack thereof) was used to cluster the sequences in question (SS = Salmo Salar, ST = Salmo Trutta, and CG = Cogent algorithm). Following the species identifier, the SeqID contains a number indicating a locus, followed by a second number providing a unique identifier for all transcripts placed on the same locus.

Additionally, the flag -p can be used to give search results a shared prefix, aiding in the organization of search results, and the flag -c can be used to provide a different linguistic complexity cutoff (CT value described in the section above) if more or less complex motifs are desired. Lastly, the different parts of the outputs are organized into different sections (3′UTR sequence, Gene and GO annotation, UTRscan motifs, Teiresias Motifs, Target Prediction, and Target Summary), which can each be selectively removed from the output files if a slimmer results file is desired. The full list of these flags for each script can be retrieved by calling the help file for each script using the flag -h. Examples of these sections are shown in Figure 2 in Section 2.1.2 of the results. All details concerning files in MicroSalmon and how to search this resource are described in the readme file in the MicroSalmon repository (http://github.com/AndreassenLab/MicroSalmon/, uploaded 13 September 2021).

## Figures and Tables

**Table 1:** Counts, descriptions, and references of all UTRscan motifs identified in at least one 3′UTR.

UTRscan Identifier	Motif Name	Total Occurrences ^1^	Unique Transcripts ^2^	Different Loci ^3^	Description	References
U0001	HSL3	5	3	2	Histone 3′UTR stem-loop structure	[30,31]
U0002	IRE	185	138	49	Iron-responsive element	[32,33,34]
U0003	SECIS1	861	857	294	Selenocysteine insertion sequence—type 1	[35,36,37,38,39,40,41,42,43,44,45,46,47]
U0004	SECIS2	786	784	266	Selenocysteine insertion sequence—type 2	[35,36,37,38,39,40,41,42,43,44,45,46,47]
U0006	CPE	4685	4685	1633	Cytoplasmic polyadenylation element	[48,49,50]
U0007	TGE	179	175	49	TGE translational regulation element	[51]
U0009	15-LOX-DICE	82	82	36	15-Lipoxygenase differentiation control element	[52,53,54]
U0010	ARE2	108	108	49	AU-rich class-2 element	[55]
U0012	GLUT1	79	79	33	Glusose transporter type-1 3′UTR cis-acting element	[56]
U0016	SXL_BS	6495	5941	2123	SXL binding site	[57,58,59,60,61]
U0017	UNR-bs	5883	5420	2039	UNR binding site	[62,63,64,65,66,67,68,69,70,71,72]
U0019	BRE	355	345	122	Bruno 3′UTR responsive element	[73,74]
U0020	ADH-DRE	1529	1461	556	Alcohol dehydrogenase 3′UTR downregulation control element	[75,76]
U0022	PRONEURAL-BOX	1	1	1	Proneural box	[77,78]
U0023	K-BOX	17188	14341	5159	K-box	[79,80]
U0024	BRD-BOX	7279	6635	2319	Brd-box	[79,80]
U0025	GY-BOX	4990	4433	1693	GY-box	[78,79,80]
U0027	G3A	11	11	3	Elastin G3A 3′UTR stability motif	[81]
U0028	INS_SCE	14	14	6	Insulin 3′UTR stability element	[82]

^1^ Total number of observations of the motif in the 3′UTRome; ^2^ total number of transcripts with one or more occurrences of this motif in their 3′UT; ^3^ total number of different loci from where transcripts containing this motif in their 3′UTR were derived. Annotation of loci was based on the SeqIDs in Ramberg et al. [24].

**Table 2:** Nine genes with IRE motifs and GO terms or other annotation associated with iron metabolism.

Iron-Relevant GO Terms ^1^	Manual Gene Annotation ^2^
Iron binding	timm2
Iron ion homeostasis	meltf
Transferrin receptor	trfc paralog 1
Transferrin receptor	trfc paralog 2
**—** ^3^	qsox1
Metalloreductase	steap3
Iron ion binding	agmo
Heme binding	dgcr8
Iron ion binding	fa2h

^1^ Gene Ontology term annotation of these transcripts in Ramberg et al. [24]; ^2^ manual annotation of genes, as described in the Materials and Methods section, to replace generic gene IDs with gene symbols. This was necessary to facilitate gene pathway enrichment analysis; ^3^ LOC106613912 was manually annotated as qsox1, a gene regulated by the IRE motif and coding for an iron-responsive tissue-remodeling factor in higher vertebrates [86,87].

**Table 3:** Distribution of over-represented sequence motifs with lengths from 7 to 16 nts.

Motif length ^1^	7	8	9	10	11	12	13	14	15	16
All motifs ^2^	1	81	230	199	59	19	9	3	2	1
Motifs containing a seed sequence ^3^	0	4	36	34	6	2	0	0	0	0
Complexity filter motifs (CT > 0.27) ^4^	0	3	44	33	2	0	0	0	0	0
Filtered motifs containing a seed sequence ^5^	0	1	9	4	0	0	0	0	0	0

^1^ The length (nts) of the over-represented motifs; ^2^ number of over-represented motifs in each size group (Teiresias analysis results); ^3^ number of over-represented motifs containing a seed-target as a subsequence; ^4^ number of remaining over-represented motifs after complexity filtering (CT > 0.27); ^5^ number of over-represented complexity-filtered motifs containing a seed-target as a subsequence.

## Data Availability

The datasets presented in this study are found in the Supplementary Files and under NCBI Bioproject PRJNA680991 and the MicroSalmon GitHub repository at http://github.com/AndreassenLab/MicroSalmon/ (Uploaded 13 September 2021). Inhouse Python scripts used for analysis are available upon request.

## Supplementary Materials

The following are available online at https://www.mdpi.com/article/10.3390/ncrna7040061/s1, File S1: Example search output for mRNA search script, File S2: Example search output for miRNA search script, File S3: Automated and manual annotation of transcripts with IRE motifs, File S4: Over-represented Teiresias motifs containing miRNA seed target sequences, File S5: Sequence and position distributions for all over-represented Teiresias motifs, File S6: Fasta file containing PAS motifs and the 54 nt upstream sequence for 100 transcripts, File S7: Comparison of predicted targets from the SAV study [27] to the predicted targets in MicroSalmon using the same miRNAs as input.
